# Quantum formalism for the dynamics of cognitive psychology

**DOI:** 10.1038/s41598-023-43403-4

**Published:** 2023-09-26

**Authors:** Dorje C. Brody

**Affiliations:** School of Mathematics and Physics, University of Surrey, Guildford, GU2 7XH UK

**Keywords:** Quantum mechanics, Cognitive neuroscience

## Abstract

The cognitive state of mind concerning a range of choices to be made can be modelled efficiently by use of an element of a high-dimensional Hilbert space. The dynamics of the state of mind resulting from information acquisition can be characterised by the von Neumann–Lüders projection postulate of quantum theory. This is shown to give rise to an uncertainty-minimising dynamical behaviour equivalent to Bayesian updating, hence providing an alternative approach to representing the dynamics of a cognitive state, consistent with the free energy principle in brain science. The quantum formalism, however, goes beyond the range of applicability of classical reasoning in explaining cognitive behaviour, thus opening up new and intriguing possibilities.

## Introduction

The present paper is concerned with the use of Hilbert space techniques, so successfully implemented in characterising behaviours and properties of quantum systems^1^, to model cognitive ‘psychology’ in the sense to be defined below. The paper, on the other hand, is *not* concerned with whether quantum effects seen in microscopic physical systems, such as interference, violation of the Bell inequality, or the use of complex numbers, might play a role in psychology or in brain activity. There are differing opinions on the matter^2–7^, but these will not be addressed here, for, the use of Hilbert space techniques in modelling cognitive behaviour, in itself, does not necessarily require the actual functioning of the brain’s neurophysiology to be quantum mechanical. What does concern us will include the tensor product structure of the Hilbert space, entanglements, the superposition principle, the projection postulate, and decoherence. While some may view these to be intrinsically quantum-mechanical effects, I shall show that they are in fact intrinsic to any probabilistic system modelled on a Hilbert space—an idea that is rooted in the pioneering work of Rao^8^ (see^9^ for a related discussion). In any case, my purpose here is to illustrate how it is both natural and effective to implement Hilbert space techniques in modelling human cognitive behaviour. Such a proposal, in itself, is not new (see, e.g.,^10–13^ and references cited therein). My main contribution is to introduce a formalism that allows for a systematic treatment of the *dynamics* of the cognitive state of mind, from which predictions can be made about human behaviour.

The key idea to be explored is that the state of mind of a person, to be defined more precisely in what follows, can be represented efficiently in terms of a vector in a high-dimensional Hilbert space, which in turn is a tensor product of lower-dimensional Hilbert spaces. However, before turning to technical discussions, I would like to illustrate the significance of the superposition principle in this context through simple examples. The first concerns the tossing of a fair coin. When a coin is tossed, but the outcome not yet revealed, no one will dispute the state of the coin, which is a macroscopic classical object: it is *either* in the ‘heads’ state *or* in the ‘tails’ state. In fact, even before the coin is tossed, it will be known to all that the state of the coin *will be* either heads or tails. However, a person who is in the position of guessing the outcome will have a different state of mind. Until the moment when a choice is made, the person’s mind is *neither* in a state of heads *nor* in a state of tails—it is in a state of superposition, to be explained below. Thus there is a dissonance between the objective reality of the state of the coin and the state of the mind attempting to determine the state of the coin.

Another example is seen in children’s hand game of rock, scissors and paper. Again, until the last moment when the decision is made as to which hand to choose, the person’s mind is in the state of a superposition between the three alternatives. Of course, with any decision, a person may come with a predetermined outcome – as in Groucho Marx’s, “Whatever it is, I’m against it!” But until when that decision is made, the fact remains that the mind is in an indefinite state. Only at the moment when the decision is made, for example to declare that the outcome of the coin tossing will be heads, or choosing the scissors hand, the state ‘collapses’ into one of the definite states. This situation is very much analogous to the measurement process in quantum theory.

With these heuristics in mind, the present paper will be organised as follows. I begin by defining what I mean by the state of mind, which will essentially be a Hilbert-space representation of the probability assignments to the totality of the choices available to the person. A given set of choices will then be modelled as an observable acting on this Hilbert space. The idea I put forward here, which is part of an on-going programme initiated in^14^, is closely related to the proposals made by Yukalov and Sornette^12^, Busemeyer and his collaborators^15^, and Khrennikov and his collaborators^16^. In my approach I model the dynamics of cognitive behaviour directly using a quantum formalism that takes into account the impact of noise, rather than starting from the quantum theory and then deducing implications in behavioural modelling by reduction. As a consequence, I am able to gain certain insights into human behaviour that go beyond what is commonly understood in cognitive psychology. I examine how the state of mind changes when a person acquires noisy information that is relevant to decision making. My approach will be consistent with the cybernetics framework of Wiener as an attempt to understand the dynamics of living systems^17^. I use the von Neumann-Lüders projection postulate in quantum mechanics to arrive at the change in the state of mind, and show that the result agrees with the classical formulation using the Bayes formula; in agreement with related previous findings^16^. Further, I show that the projection postulate gives rise to an evolution that on average minimises future ‘surprises’, and hence is consistent with the free-energy principle widely adopted in brain science^18^. I then consider what happens when noisy information arrives in continuous time. When the dynamical evolution of the state of mind resulting from a sequential von Neumann-Lüders projection postulate is reduced to a projective Hilbert space, the dynamical equation reveals an important feature, namely, that each state of zero uncertainty, having vanishing Shannon-Wiener entropy, acts as an attractor to the dynamics. The result provides a geometric explanation of certain characteristics of human behaviour seen in psychology literature (such as confirmation bias), which I have previously called the tenacious Bayesian behaviour^19^. The work presented here demonstrates the fact that if a person’s opinion is strongly skewed towards one of the false alternatives, and if all choice observables are commutative, then even if partial information about the truth is revealed and the person behaves rationally in accordance with Bayesian updating, it is very difficult for the person to escape from the initial misconception. In other words, adherences to false narratives commonly observed in today’s society need not be due to irrational behaviour. Following the proposal in^10^, I then consider what happens if choice observables are not commutative. This is motivated by the observation that many empirical observations concerning cognitive human behaviour cannot be fully described by use of a commuting set of observables. It will be shown that the existence of incompatible observables can be used to rescue a person fallen into a false attractor—a possibility that is unavailable by use of purely classical reasonings.

## Decision making and the state of mind

Throughout the paper I shall be concerned with the cognitive process of decision making, which will be a topic distinct from what is known as statistical decision theory, for which there are excellent treatises^20,21^. Decision making occurs when a person is unsure from which alternative to choose; but I will be using the term ‘decision making’ in a broad sense to include a person’s uncertain point of view on a topic, for which there are multiple views, and for which there may not be a need to choose one particular alternative. In any case, this uncertainty, which is largely due to lack of sufficient information, can be modelled in the form of a set of probabilities that represents the likelihoods of different alternatives being selected. Suppose that a decision needs to be made to choose one out of *N* alternatives. The number of alternatives can be infinite, or even uncountable—the formalism extends straightforwardly to these cases, but for simplicity I consider the finite case here. That said, my own view is that the use of infinite or uncountable variables is merely for mathematical convenience, for, the reduction of uncertainty in determining the value of a continuous variable requires infinite information acquisition, which is not available in real world. At a given moment in time, let $p_k$ denote the likelihood that the *k*th alternative is selected. If $p_k=1$ for a value of *k* then the mind is in a definite state in relation to this decision. Hence for a given decision, the set of numbers $(p_1,p_2,\ldots ,p_N)$ represents the state of mind in relation to that decision making.

It will be useful here to explain in more detail the meaning of these probabilities. For this purpose, consider the example, say, of choosing an item from a menu in a restaurant. The likelihood of selecting a given choice by a person, in fact, cannot be inferred, because in general a repeated “measurement” is not permissible in cognitive psychology. This follows from the fact that after an experiment, information is typically acquired to alter the state of mind of the person (e.g., “I have tried this dish and it was not so nice.”). Hence in general one cannot reconstruct the state of mind of a person on a given choice. This is entirely analogous to the situation in quantum mechanics whereby in general one cannot perform a measurement without altering the state of the system.

To overcome this issue, consider, hypothetically, a large number of identical “clones” of the person, all of whom are faced with the same decision at the same time. The fact that there are uncertainties in the choice means that different clones will make different choices, the results of which can then be used to infer the likelihoods of different alternatives being chosen. This is essentially the idea of an “ensemble” introduced by Gibbs. Of course in reality there are no clones. In cognitive psychology the issue is handled by performing instead experiments on a large number of people. Each person is in a different state of mind, but if the group of people have some level of commonality, then they can be viewed as clones of a hypothetical representative of the group.

At any moment in time one faces a multitude of decisions, not just one, some of which are intertwined with each other while others are independent. In probability theory, such a situation is modelled by means of a joint probability for the totality of decisions. Alternatively, the situation can be modelled on a Hilbert space by use of the square-root map: $p_k \rightarrow \psi _k = \sqrt{p_k}$. Here by a Hilbert space ${{\mathcal {H}}}$, I mean a real vector space endowed with a Euclidean inner product, such that each element of ${{\mathcal {H}}}$ has a finite norm. A typical element of ${\mathcal {H}}$, i.e. a real vector of finite length, is denoted $|\psi \rangle$, whose transposition is written $\langle \psi |$ in the usual Dirac notation. Thus the inner product of a pair of elements $|\psi \rangle$ and $|\phi \rangle$ in ${{\mathcal {H}}}$ is written

$$\begin{aligned} \langle \phi |\psi \rangle = (\phi _1 \, \phi _2 \, \cdots \, \phi _N) \left( \begin{array}{c} \psi _1 \\ \psi _2 \\ \vdots \\ \psi _N \end{array} \right) = \sum _{i=1}^N \phi _i \psi _i. \end{aligned}$$

An element $|\psi \rangle \in {{\mathcal {H}}}$, which is a vector, can also be expressed in the matrix form as follows:

$$\begin{aligned} |\psi \rangle \langle \psi | = \left( \begin{array}{c} \psi _1 \\ \psi _2 \\ \vdots \\ \psi _N \end{array} \right) (\psi _1 \, \psi _2 \, \cdots \, \psi _N) = \left( \begin{array}{cccc} \psi _1^2 &{} \psi _1 \psi _2 &{} \cdots &{} \psi _1 \psi _N \\ \psi _2\psi _1 &{} \psi _2^2 &{} \cdots &{} \psi _2\psi _N \\ \vdots &{} \vdots &{} \ddots &{} \vdots \\ \psi _N\psi _1 &{} \psi _N\psi _2 &{} \cdots &{} \psi _N^2 \end{array} \right) . \end{aligned}$$

It will be shown that the norm of a vector carries no psychologically relevant information. Thus, in what follows it will be assumed that all vectors have unit norm. Clearly, the vector with components $\{\psi _k\}$, in the basis $|e_k\rangle = (0,0,0,\ldots ,1,0,\ldots ,0)$, with only the *k*th element nonzero, is an element of an *N*-dimensional real Hilbert space ${{\mathcal {H}}}^N$. Thus, in the Dirac notation the state can be expressed in the form of a superposition $|\psi \rangle = \sum _k \psi _k |e_k\rangle$. If there is a second decision to be made out of *M* alternatives, then the state of a person’s mind in relation to these two choices is represented by an element of the tensor product ${{\mathcal {H}}}^N\otimes {{\mathcal {H}}}^M$. This tensor product structure arises solely from statistical dependencies of two decisions, when modelled on a Hilbert space.

There are reasons for choosing to work with a Hilbert space ${{\mathcal {H}}}$ of square-root probability vectors, rather than the probabilities themselves. To this end recall the example of a person who is attempting to guess the outcome of a coin toss. I have indicated that the physical state of a tossed coin is that of either heads or tails, and this is represented on ${{\mathcal {H}}}$ by what is called a *mixed state*. Writing $|1\rangle$ for the ‘heads’ state and $|0\rangle$ for the ‘tails’ state, and *p* for the bias of the coin, the ‘either-or’ state is given by the following mixed-state density matrix:

$$\begin{aligned} {\hat{\rho }} = p|1\rangle \langle 1| + (1-p)|0\rangle \langle 0|. \end{aligned}$$

The state of mind of a person trying to guess the outcome, on the other hand, is neither heads nor tails, and this is represented on ${{\mathcal {H}}}$ by a linear superposition

$$\begin{aligned} |\psi \rangle = \sqrt{p} \, |1\rangle + \sqrt{1-p} \, |0\rangle , \end{aligned}$$

which can equally be represented in the form of a *pure-state* density matrix $|\psi \rangle \langle \psi |$. The two states ${\hat{\rho }}$ and $|\psi \rangle \langle \psi |$ are different. Working with the mixed state ${\hat{\rho }}$ is equivalent to working with classical probabilities, whereas in general the properties of a pure state $|\psi \rangle$ cannot be fully captured using the language of classical probabilities. Of course, if the only decision at hand concerns the guessing of the outcome of a coin toss, then there are no psychological experiments one can perform that will distinguish the states ${\hat{\rho }}$ and $|\psi \rangle$. That is, with merely one decision it is not possible to determine statistically, even if a large number of clones are available, whether the state of mind of a person is that of ‘either-or’ or ‘neither-nor’. This makes it legitimate to work with classical probabilities when dealing with a single decision. However, if there are more decisions involved, and if some of the decisions are not compatible in the sense explained below—and there are ample empirical data suggesting some decisions are not compatible^22^—then it is possible to experimentally distinguish the mixed state ${\hat{\rho }}$ from the pure state $|\psi \rangle$. In this situation, the use of Hilbert space techniques becomes a necessity, because cognitive behaviour resulting from incompatible decisions cannot in general be modelled by use of classical probabilistic reasonings, but can be explained by use of quantum probabilities^11^.

The Hilbert-space construction outlined here extends to the case where an arbitrary number of decisions are to be made. With this in mind, I define the state of mind of a person facing a range of alternatives to consider, at any moment in time, to be an element of the tensor product ${{\mathcal {H}}}=\otimes _{l=1}^K{{\mathcal {H}}}_l$, where *K* is the number of distinct decisions. If two decisions can be made independently, then the component of the state vector belonging to the corresponding subspace of ${{\mathcal {H}}}$ will be in a product state. Otherwise, a state is entangled. As a simple example, consider a pair of binary decisions, for example, whether to take fish or meat for the main course, and whether to take red or white wine to accompany it. Writing $|F\rangle$ and $|M\rangle$ for the food choices, and similarly $|R\rangle$ and $|W\rangle$ for the wine selections, if the state of mind of a person is $|\psi \rangle = c_1 |FW\rangle + c_2 |MR\rangle$, then the person will choose fish with white wine with probability $c_1^2$, and meat with red wine with probability $c_2^2=1-c_1^2$; but no other option will be chosen. This is evidently an entangled state (a state that cannot be expressed in the form of $|XY\rangle$, where $|X\rangle$ is an arbitrary linear combination of $|F\rangle$ and $|M\rangle$, and $|Y\rangle$ is an arbitrary linear combination of $|R\rangle$ and $|W\rangle$), which collapses to one or the other alternatives at the moment the waiter arrives and takes the order.

In this Hilbert space formulation, a given choice can be modelled by a real symmetric matrix, whose dimension is the number of alternatives. Such a matrix corresponds to observables in quantum mechanics. I will assume, for now, that all such ‘observables’ or ‘choices’ are compatible in the sense that the matrix representations can be diagonalised simultaneously. What this means is that at any given time, an arbitrary number of decisions can be made simultaneously. It is then evident that no state of mind, whether entangled or not, can violate laws of classical probability, and hence no state can violate, in particular, Bell’s inequalities. Later in the paper, however, I will consider the case where choice observables are incompatible.

The eigenvalues of the choice observables then label different alternatives. This is analogous to quantum observables when it concerns labelling outcomes of a single measurement. However, observables in quantum theory have a second role apart from representing measurement outcomes: they generate dynamics. As a consequence, the differences of observable eigenvalues have direct physical consequences, and hence they cannot be chosen arbitrarily. It appears, in contrast, that the differences of eigenvalues of the choice observables have no significance: the results of a selection, such as choosing a hand in the game of rock, scissors and paper, can be labelled by means of any three distinct numerical values, merely as place keepers so that statistical analysis can be applied. It will be shown below, however, that when it concerns the dynamics of the state of mind, the eigenvalue differences do play an important role, and hence, just as in quantum theory, they cannot be chosen arbitrarily,

It may be helpful to remark here about the use of *real* Hilbert spaces in the present consideration, as opposed to complex Hilbert spaces required for the characterisation of quantum systems. In quantum theory, an imaginary number (or a complex structure, more precisely) plays two roles: one concerns the rotation of a plane wave by the right angle, and the other concerns the identification of the orientation of the time axis, connected to the unitary time evolution. To my knowledge in cognitive psychology there has been no phenomenon identified that suggests the requirement of a complex number—perhaps because this is not a question that has emerged in the past. Hence, although it seems plausible that complex numbers may ultimately be required for a more adequate characterisation of cognitive behaviour, for now I shall confine my analysis to real Hilbert space.

## Dynamics

Having established the framework for representing the cognitive state of mind, let us explore how the state changes in time. To this end I shall be working under the hypothesis that a given state $|\psi \rangle$ of a person’s mind changes only by transfer of information. It is, of course, possible that an isometric motion analogous to the unitary motion of quantum theory that does not exchange information can change the state (for example, to start wondering about the feasibility of a choice immediately after making the choice without any external information), and if so this will be given by an orthogonal transformation. However, without any clear physical or psychological evidence indicating the existence of such a symmetry, I shall not consider this possibility, and focus instead on the universally acknowledged empirical fact that information acquisition (or information loss) will inevitably change the state of a mind. The question is, in which way?

To understand dynamics, I shall be borrowing some ideas from communication theory. Focusing on a single decision to start with, let ${{\hat{X}}}$ denote the decision or choice observable, with eigenvalues $\{x_k\}_{k=1,\ldots ,N}$. These eigenvalues for now merely label different alternatives. The eigenstate $|x_j\rangle$ of ${{\hat{X}}}$, satisfying ${{\hat{X}}}|x_j\rangle =x_j|x_j\rangle$, thus represents the state of mind in which the *j*th alternative has been chosen. Prior to an alternative being chosen, the state is in a superposition $|\psi \rangle = \sum _k c_k |x_k\rangle$. The state will change when the person acquires information relevant to the decision making. This information is rarely perfect. In communication theory, anything that obscures finding the value of the quantity of interest is modelled in terms of noise. Let ${\hat{\varepsilon }}$ denote this noise. Here, ${\hat{\varepsilon }}$ can take discrete values, or more commonly continuous values. I will consider the latter case so that ${\hat{\varepsilon }}$ acts on an infinite dimensional Hilbert space ${{\mathcal {H}}}^\infty$ distinct from the state space ${{\mathcal {H}}}^N$. The noise arises from external environments ${{\mathcal {E}}}$. For simplicity I shall assume that the state of noise is pure, and is given by $|\eta \rangle =\eta (y) \in {{\mathcal {H}}}^\infty$, although a mixed state can equally be treated. Then initially the state of mind of a person attempting to make a decision and the state of the noise-inducing environment as perceived by the decision maker are disentangled, and when taken together can be represented by the product state $|\psi \rangle |\eta \rangle$.

Acquisition of partial information relevant to decision making can be modelled by observing the value of

$$\begin{aligned} {\hat{\xi }} = {{\hat{X}}} + {\hat{\varepsilon }}. \end{aligned}$$

   Here, the sum is taken in the tensor-product space ${{\mathcal {H}}}^N\otimes {{\mathcal {H}}}^\infty$. To understand this composite sum, consider the case in which ${\hat{\varepsilon }}$ is finite and can take three values $\varepsilon _1,\varepsilon _2,\varepsilon _3$, while the decision is binary, represented by the values $x_0$ and $x_1$. Then ${\hat{\xi }}$ is a $6\times 6$ matrix with the eigenvalues $x_0+\varepsilon _1$, $x_0+\varepsilon _2$, $x_0+\varepsilon _3$, $x_1+\varepsilon _0$, $x_1+ \varepsilon _1$, $x_1+\varepsilon _3$. In general, the eigenvalues of ${\hat{\xi }}$ are highly (typically *N*-fold) degenerate. The form that ${\hat{\xi }}$ takes is of course nothing more than a signal-plus-noise decomposition in classical communication theory^17^. The ‘signal’ term, more generally, will be a function $F({{\hat{X}}})$ of ${{\hat{X}}}$, but for simplicity I shall assume the function to be linear because the choice of *F*(*x*) is context dependent.

Once the value of the information-providing observable ${\hat{\xi }}$ is measured, the initially-disentangled state

$$\begin{aligned} |\psi \rangle |\eta \rangle = \sum _k \sqrt{p_k} \, \eta (y) |x_k\rangle \end{aligned}$$

becomes an entangled state. In quantum mechanics, the transformation of the state after measurement is given by the von Neumann-Lüders projection postulate. That is, writing

$$\begin{aligned} {\hat{\Pi }}_\xi = \sum _k \delta (y-\xi +x_k) |x_k\rangle \langle x_k| \end{aligned}$$

for the projection operator onto the subspace of ${{\mathcal {H}}}^N\otimes {{\mathcal {H}}}^\infty$ spanned by the eigenstates of ${\hat{\xi }}$ with the eigenvalue $\xi$, the projection postulate asserts that the state of the system after information acquisition is

$$\begin{aligned} {\hat{\rho }}_\xi = \frac{{\hat{\Pi }}_\xi |\psi \rangle |\eta \rangle \langle \psi |\langle \eta |{\hat{\Pi }}_\xi }{\textrm{tr}\left( {\hat{\Pi }}_\xi |\psi \rangle |\eta \rangle \langle \psi |\langle \eta |{\hat{\Pi }}_\xi \right) }. \end{aligned}$$

A short calculation then shows that this is given more explicitly by

$$\begin{aligned} {\hat{\rho }}_\xi = \frac{\sum _{k,l} \sqrt{p_k p_l} \, \eta (\xi -x_k) \eta (\xi -x_l) \, |x_k\rangle \langle x_l|}{\sum _m p_m \eta ^2(\xi -x_m)}. \end{aligned}$$

Two interesting observations that follow are in order. First, the density matrix by construction is a projection operator onto a pure state $|\psi (\xi )\rangle$ given by

$$\begin{aligned} |\psi (\xi )\rangle = \sum _k \sqrt{\pi _k(\xi )}\, |x_k\rangle , \end{aligned}$$

where $\xi$ is the value of the detected signal. Second, the coefficients of the pure state agrees with the conditional probability of the choice given by the Bayes formula:

$$\begin{aligned} \pi _k(\xi ) = \frac{p_k\,\eta ^2(\xi -x_k)}{\sum _m p_m \eta ^2(\xi -x_m)}. \end{aligned}$$

That is, $\pi _k(\xi )$ is the probability that the *k*th alternative is chosen, conditional on observing the value $\xi$ of ${\hat{\xi }}$. It follows that the von Neumann-Lüders projection postulate of quantum theory not only gives the correct classical result (as already observed in^7,16^ with a different construction) but also provides a simple geometric interpretation of the Bayes formula. This follows because the Lüders state $|\psi (\xi )\rangle$ associated to a degenerate measurement outcome $\xi$ is given by the orthogonal projection of the initial state onto Hilbert subspace associated to this outcome. Hence $|\psi (\xi )\rangle$ is the closest state on the constrained subspace in terms of the Bhattachayya distance^23^ to the initial state $|\psi \rangle |\eta \rangle$.

It is worth remarking that an alternative interpretation of the von Neumann-Lüders projection postulate can be given in terms of the so-called free energy principle^24,25^. Intuitively, this principle asserts that the change in the state of mind follows a path that on average minimises elements of surprise. In the present context, the degree of surprise can be measured in terms of the level of uncertainty. Suppose that the state of mind after information acquisition becomes ${\hat{\mu }}_\xi$ that is different from the Lüders state ${\hat{\rho }}_\xi$. Then the level of uncertainty associated with the choice observable ${{\hat{X}}}$ resulting from ${\hat{\mu }}_\xi$, conditional on the observed value $\xi$ of ${\hat{\xi }}$, is given by

$$\begin{aligned} \textrm{tr}\left( \left[ {{\hat{X}}}-\textrm{tr}({{\hat{X}}}{\hat{\mu }}_\xi )\right] ^2{\hat{\rho }}_\xi \right) = \textrm{tr}\left( {{\hat{X}}}^2{\hat{\rho }}_\xi \right) - \left( \textrm{tr}\left( {{\hat{X}}}{\hat{\rho }}_\xi \right) \right) ^2 + \left( \textrm{tr}\left( {{\hat{X}}}{\hat{\delta }}_\xi \right) \right) ^2, \end{aligned}$$

where I have written ${\hat{\delta }}_\xi ={\hat{\mu }}_\xi -{\hat{\rho }}_\xi$ for the deviation. Since the first two terms on the right hand side together constitute the conditional variance of ${{\hat{X}}}$, which is positive and is independent of ${\hat{\mu }}_\xi$, to minimise the expected uncertainty, and hence the surprise, for all ${{\hat{X}}}$ and $\xi$, it has to be that ${\hat{\delta }}_\xi =0$. It follows that among all the states consistent with the observation, the Lüders state is unique in that it minimises the expected level of future surprise, as measured by the uncertainty.

I might add parenthetically that a psychologist wishing to predict the statistics of the behaviour of a person who has acquired information relevant to decision making will *a priori* not know the observed value of $\xi$. To a psychologist, $\xi$ is thus a random variable with the density $p(y)=\sum _m p_m \eta ^2(y-x_m)$. That is, given the state $|\psi \rangle |\eta \rangle$, the probability of the measurement outcome of the observation of ${\hat{\xi }}$ lying in the interval $[y,y+\textrm{d}y]$ is given by $p(y)\textrm{d}y$. Hence, in this case the density matrix ${\hat{\rho }}_\xi$ has to be averaged over $\xi$, but the denominator of ${\hat{\rho }}_\xi$ is just the density *p*(*y*) for $\xi$, so the averaged density matrix is given by

$$\begin{aligned} {{\mathbb {E}}}[{\hat{\rho }}_\xi ] = \sum _{k,l} \sqrt{p_k p_l} \, \Lambda (\omega _{kl}) \, |x_k\rangle \langle x_l|, \end{aligned}$$

where $$\omega _{kl}=x_k-x_l$$ and

$$\begin{aligned} \Lambda (\omega ) = \int _{-\infty }^\infty \eta (y) \eta (y-\omega ) \textrm{d}y. \end{aligned}$$

Evidently, $0\le \Lambda (\omega _{kl})\le 1$ and $\Lambda (\omega _{kk})=1$ for all *k*, *l*, but because the initial state of mind $|\psi \rangle \langle \psi |$ in this basis has the matrix elements $\{\!\sqrt{p_kp_l}\}$, it follows that an external observer (e.g., a psychologist) will perceive a decoherence effect whereby the off-diagonal elements of the reduced density matrix are damped.

It is at this point that I wish to comment on the numerical values of the differences $\{\omega _{kl}\}$. While there is no reason why $\Lambda (\omega )$ should be monotonic in $\omega$ (unless $\eta (y)$ is unimodal), it will certainly be the case that the decoherence effect is more pronounced for large values of $\omega$. That is, $\Lambda (\omega )\ll 1$ for $\omega \gg 1$. For the same token, the values of $\omega _{kl}$ will directly affect the conditional probabilities $\{\pi _k(\xi )\}$. Therefore, while the values of $\omega _{kl}$, and hence those of $x_k$, can be chosen arbitrarily to describe the statistics of the initial state of mind, once the dynamics have been taken into account (what happens *after* information acquisition), it becomes evident that they cannot be chosen arbitrarily.

A better intuition behind this observation can be gained by reverting back to ideas of signal detection in communication theory. For this purpose, consider a binary decision. Supposed that the eigenvalues of ${{\hat{X}}}$ labelling the two decisions are chosen to be, say, $\pm 0.1$ and suppose that the noise distribution $\eta ^2(y)$ is normal, centred at zero, with a small standard deviation. In this case, the observed outcomes of ${\hat{\xi }}$ will most likely take values close to zero. As a consequence, a single observation of ${\hat{\xi }}$ will reduce on average the initial uncertainty only by a very small amount. In contrast, suppose that the two eigenvalues of ${{\hat{X}}}$ are chosen to be $\pm 10$, but the noise is the same as before. Then the observation will almost certainly yield the outcome that is close to $+10$ or $-10$. Hence the uncertainty in this case has been reduced to virtually zero after a single observation. This extreme example shows how it is not possible to label different choice alternatives by arbitrary numerical numbers, while at the same time adequately modelling the dynamics of the state of mind.

In the event where a model $\eta (y)$ for the state of noisy environment exists, it is possible in principle to estimate the eigenvalue differences $\{\omega _{ij}\}$ by studying how much a person’s views shifted from the acquisition of the noisy information. This is because the average reduction of uncertainty, as measured by entropy change or the decoherence rate, is determined by the eigenvalue differences $\{\omega _{ij}\}$. The implication of the choices of the eigenvalue differences $\{\omega _{ij}\}$ on voter behaviours in an electoral competition has recently been examined^26^.

## Sequential updating

I have illustrated how the cognitive state of mind of a person in relation to a given choice changes after a single acquisition of information. A more interesting, as well as realistic, situation concerns the sequential updating of the state of mind as more and more noisy information is revealed. In this case the information-providing observable ${\hat{\xi }}_t$ is a time series. As a simple example that extends the previous one very naturally, I consider the time series

$$\begin{aligned} {\hat{\xi }}_t = {{\hat{X}}}t+{\hat{\varepsilon }}_t, \end{aligned}$$

where the noise term ${\hat{\varepsilon }}_t$ is modelled by a standard Brownian motion $\{B_t\}$ multiplied by the identity operator of the Hilbert space ${{\mathcal {H}}}^\infty$. The ‘signal’ component, more generally, can be given by $\int _0^t F_s({{\hat{X}}}) \textrm{d}s$, but again for simplicity I assume that the function $F_t(x)$ is linear for all *t*. In fact, even more generally, the range of alternatives ${{\hat{X}}}$ itself can be time dependent, but I do not consider this case here.

In this example, what happens to the state of mind can be worked out by discretising the time interval [0, *t*] and taking the limit. Starting from time zero, over a small time increment $\textrm{d}t$ the initial state $|\psi \rangle |\eta \rangle$ is projected to the Lüders state ${\hat{\Pi }}_{\xi _{\rm{d}t}}|\psi \rangle |\eta \rangle$, suitably normalised, in accordance with the projection postulate. The noise is normally distributed with mean zero and variance $\textrm{d}t$, so that $\eta (y)$ is the square-root of the corresponding Gaussian density function. Then after another time interval $\textrm{d}t$ we apply the projection operator again, normalise it, and repeat the procedure until time *t*. Finally, taking the limit, a calculation shows that the Lüders state, after monitoring the observable ${\hat{\xi }}_t$ up to time *t*, is given by

$$\begin{aligned} |\psi (\xi _t)\rangle = \frac{1}{\sqrt{\Phi _t}} \sum _k \sqrt{p_k} \, \textrm{e}^{ \frac{1}{2}x_k \xi _t - \frac{1}{4} x_k^2 t}\, |x_k\rangle , \end{aligned}$$

where $\xi _t=Xt+B_t$ and *X* is the random variable represented on the Hilbert space ${{\mathcal {H}}}^N$ by the operator ${{\hat{X}}}$ along with the initial state $|\psi \rangle$; that is, *X* takes the value $x_k$ with the probability $p_k$, and $\Phi _t = \sum _k p_k \textrm{e}^{ k_k \xi _t - \frac{1}{2} x_k^2 t}$ gives the normalisation.

Since we have an explicit expression that monitors the change in the state of mind as information is revealed, there is *a priori* no reason to identify the differential equation to which $|\psi (\xi _t)\rangle$ is the solution. Nevertheless, the exercise of working out the dynamical equation provides several new insights worth discussing. The detailed mathematical steps required here to work out the dynamics has been outlined in^27^, so I shall not repeat this. It suffices to say that the Lüders state is a function of *t* and $\xi _t$, where the latter is a Brownian motion with a random drift. Hence the relevant calculus to apply is that of Ito: one Taylor expands $|\psi (\xi _t)\rangle$ in *t* and $\xi _t$, and retain leading-order terms, bearing in mind that $(\textrm{d}\xi _t)^2=\textrm{d}t$. Then it follows that

$$\begin{aligned} \textrm{d}|\psi (\xi _t)\rangle = -\frac{1}{8}({{\hat{X}}}-\langle {{\hat{X}}}\rangle _t)^2 |\psi (\xi _t)\rangle \, \textrm{d}t + \frac{1}{2} ({{\hat{X}}}-\langle {{\hat{X}}}\rangle _t) |\psi (\xi _t)\rangle \, \textrm{d}W_t, \end{aligned}$$

where $\langle {{\hat{X}}}\rangle _t=\langle \psi (\xi _t)|{{\hat{X}}}|\psi (\xi _t)\rangle / \langle \psi (\xi _t)|\psi (\xi _t)\rangle$ and where

$$\begin{aligned} \textrm{d}W_t = \textrm{d}\xi _t - \langle {{\hat{X}}}\rangle _t \, \textrm{d}t. \end{aligned}$$

The process $\{W_t\}$ defined in this way is in fact a standard Brownian motion, known as the innovations process^28^. This process has the interpretation of revealing new information. That is, while the time series $\{{\hat{\xi }}_t\}$ contains new as well as previously known information about the impending choice to be made, the process $\{W_t\}$ merely contains information that was not known previously.

There are two important observations that follow. First, the evolution of the state of mind is not directly generated by the noise $\{B_t\}$, nor by the observation $\{\hat{\xi }_t\}$. Rather, it is the innovations process that drives the dynamics. But this is the case only if the state of mind changes in such a way as to continuously minimise uncertainties. Since the expectation of the cumulative uncertainty is the entropy^27^, it follows, according to the present framework, that the tendency towards low entropy states required in biology^24^, which forms the basis of the free energy principle, emerges naturally. In particular, the implication here based on the projection postulate is that the state of mind changes only in accordance with the arrival of new information; it will not change spontaneously on its own. Second, while the analysis presented here can be deduced as a result of standard least-square estimation theory^28,29^, I have derived these results using the von Neumann–Lüders projection postulate of quantum theory. It follows that the informationally efficient dynamical behaviour of a system—efficient in the sense of minimising surprises—is applicable not only to states of mind but also to quantum systems. An analogous point of view, based on the free energy principle, has recently been proposed elsewhere^30^.

It might be added, for the purpose of psychological modelling, that the averaged reduced density matrix ${\hat{\rho }}_t={{\mathbb {E}}}[{\hat{\rho }}_{\xi _t}]$ can be seen to obey the dynamical equation

$$\begin{aligned} \frac{\partial {\hat{\rho }}_t}{\partial t} = {{\hat{X}}}{\hat{\rho }}_t{{\hat{X}}} - \frac{1}{2}\left( {{\hat{X}}}^2{\hat{\rho }}_t+{\hat{\rho }}_t{{\hat{X}}}^2 \right) . \end{aligned}$$

This, of course, is the Lindblad equation generated by the decision ${{\hat{X}}}$.

## Projecting down the dynamics

One advantage of working with the mathematical formalism of quantum theory in modelling the psychological states of mind is the deeper insight that it can uncover (cf.^10^). To this end, I note that although I have defined the state of mind as a vector in Hilbert space, what I really have in mind is a projective Hilbert space consisting of rays through the origin of Hilbert space. The idea is as follows. In probability theory, one can say, for instance, that the likelihood of an event happening is 0.3, or three out of ten, or 30%—all of these statements convey the same idea. The total probability being equal to one is merely a convenient convention that does not carry any significance. Putting it differently, working with the convention that the expectation of any decision ${{\hat{X}}}$ in a state $|\psi \rangle$ is given by the ratio $\langle {{\hat{X}}}\rangle = \langle \psi |{{\hat{X}}}|\psi \rangle /\langle \psi |\psi \rangle$, it is evident that the expectation values are independent of an overall scaling of the state $|\psi \rangle$ by a nonzero constant. Hence the Hilbert space vector $|\psi \rangle$ carries one psychologically irrelevant degree of freedom. When this degree of freedom is eliminated by identification $|\psi \rangle \sim \lambda |\psi \rangle$ for any $\lambda \ne 0$, one arrives at a projective Hilbert space, otherwise known as real projective space. This is a real manifold ${{\mathfrak {M}}}$ of dimension $N-1$, endowed with a Riemannian metric induced by the underlying probabilistic rules given by the von Neumann-Lüders projection postulate^31^.

**Figure 1:**
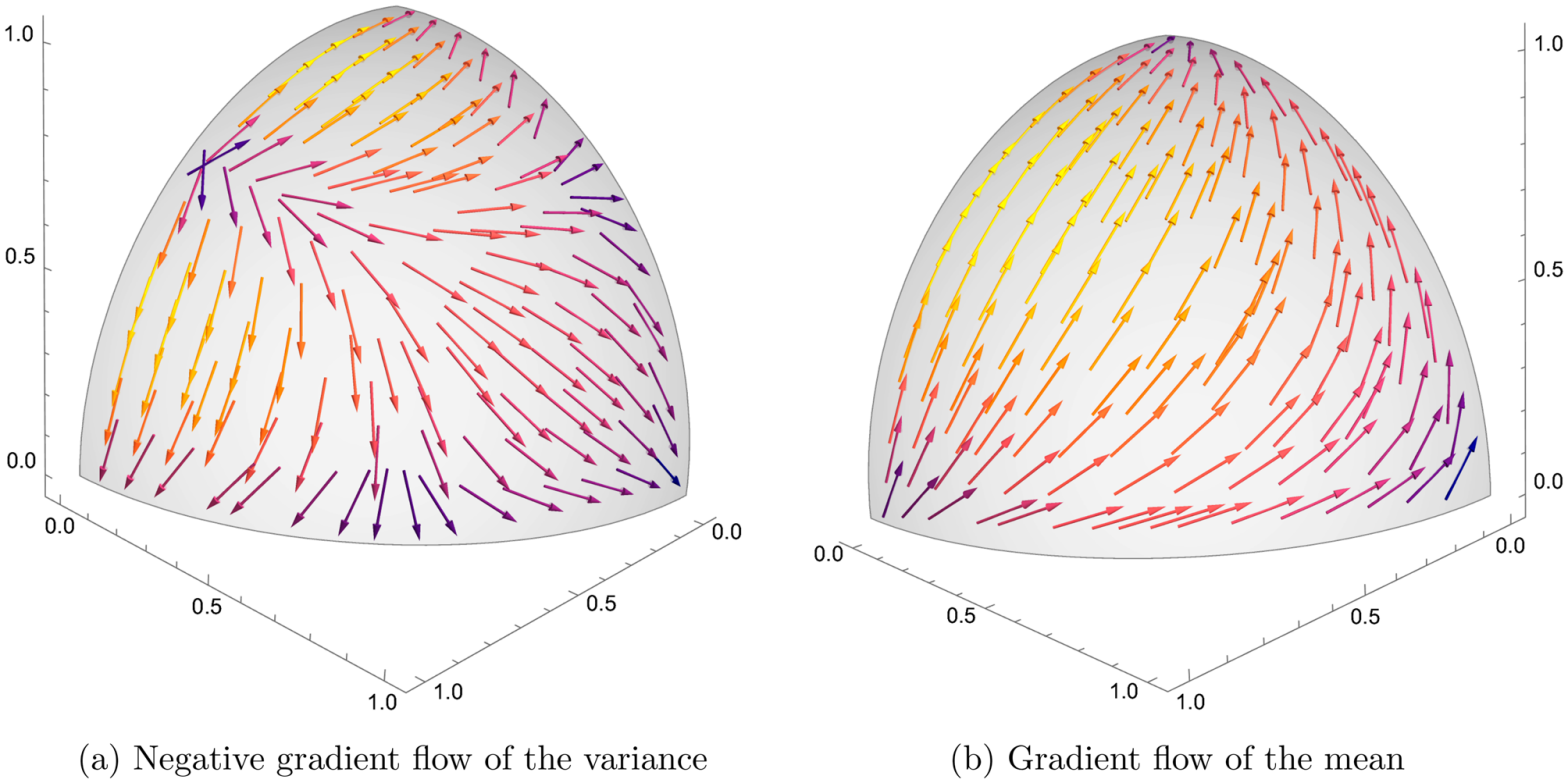
Flow of the negative gradient of the variance. In the case of a decision involving three alternatives, the corresponding state space ${{\mathfrak {M}}}$ is a real projective plane. This two-dimensional manifold is not orientable and cannot be embedded straightforwardly in three dimensions. However, it can be interpreted as a sphere with antipodal points being identified. Thus the flow on ${{\mathfrak {M}}}$ can be captured by examining the flow on the positive octant of the sphere. Here, a threefold decision is modelled by a choice observable ${{\hat{X}}}$ with eigenvalues $x_1=1$, $x_2=2$, and $x_3=3$. The three axes, corresponding to the three corners of the octant, represent the three eigenstates of ${{\hat{X}}}$ with no uncertainty. The flow $-\nabla ^a V$ generated by the negative gradient of the uncertainty (shown in the left panel) takes an initial state into one of the states with no uncertainty: this is the tendency towards least surprise. The gradient $\nabla ^a X$ of the mean is shown on the right panel for comparison. This term is multiplied by the Brownian increment $\textrm{d}W_t$, which is normally distributed with mean zero and variance $\textrm{d}t$, so at any moment in time the direction of the flow generated by the Brownian fluctuation can go either way along the flow.

Let $\{\psi ^a\}$ denote local coordinates for points on ${{\mathfrak {M}}}$. A point on ${{\mathfrak {M}}}$ thus represents a state of mind corresponding to a family of vectors $\lambda |\psi \rangle$, $\lambda \ne 0$, on Hilbert space. For any representative $|\psi \rangle$ of that family corresponding to the point $\psi \in {{\mathfrak {M}}}$, consider a function on ${{\mathfrak {M}}}$ through the expectation $X(\psi ) = \langle \psi |{{\hat{X}}}|\psi \rangle /\langle \psi |\psi \rangle$. With this convention, and writing $\nabla ^a$ for the gradient vector, the dynamical equation for the state of mind $|\psi (\xi _t) \rangle$, when projected down to ${{\mathfrak {M}}}$, is given by

$$\begin{aligned} \textrm{d}\psi ^a = - \frac{1}{16}\nabla ^a V_X \, \textrm{d}t + \frac{1}{4} \nabla ^a X \, \textrm{d}W_t, \end{aligned}$$

where $V_X(\psi )= \langle \psi |({{\hat{X}}}-X(\psi ))^2|\psi \rangle /\langle \psi |\psi \rangle$ is the function on ${{\mathfrak {M}}}$ that corresponds to the variance of ${{\hat{X}}}$ in the state $|\psi \rangle$. The mathematical analysis leading to this result have essentially been provided in^32,33^, which will not be repeated here. The important observation is that the drift term (the coefficient of $\textrm{d}t$) that generates a tendency flow on the state space ${{\mathfrak {M}}}$ is given by the negative gradient of the variance (uncertainty). Therefore, on the state space there is a tendency of driving the state of mind into one of the states with no uncertainty—this is the flow that attempts to reduce surprises. It also follows that if the state of mind is in the vicinity of one of the definite states of no uncertainty, then it will be difficult to escape from that neighbourhood—a phenomenon that I referred to as a tenacious Bayesian behaviour^19^. I shall have more to comment on this below. In Fig. 1 an example of the negative gradient flow of the uncertainty on the state space is sketched, along with the gradient flow $\nabla ^a X$ of the mean.

It is worth remarking that in principle properties of the dynamics just outlined can be inferred from the squared amplitudes

$$\begin{aligned} \pi _{kt} = \Phi _t^{-1} p_k\, \textrm{e}^{x_k \xi _t-\frac{1}{2}x_k^2 t} \end{aligned}$$

of the coefficients of $|\psi (\xi _t)\rangle$, which evidently contain as much information as the state $|\psi (\xi _t)\rangle$ itself. Indeed, starting from the expression for $\pi _{kt}$, which can be deduced by use of the Bayes formula, one can apply the Ito calculus to deduce the dynamical equation satisfied by $\pi _{kt}$, known in communication theory as the Kushner equation^34^. However, $\pi _{kt}$ is a martingale, that is, on average a conserved process. In particular, the process has no drift (the coefficient of $\textrm{d}t$ in the Kushner equation for $\pi _{kt}$ is zero), so by simply examining the Kushner equation for $\pi _{kt}$ it is difficult to infer key properties of the dynamics. In contrast, the surprise-minimising feature of the dynamics becomes immediately apparent once the process is projected to the state space ${{\mathfrak {M}}}$ via Hilbert space.

## Difference between psychological and quantum states

I have thus far emphasised the similarities in the state of mind as represented by a Hilbert space vector (or a density matrix), and the physical state of a quantum system as represented by the same scheme. There are, however, some important differences. The most important one, in my view, can be described through the following example. Suppose that the state of a quantum system is very close to one of the eigenstates $|x_k\rangle$ of an observable ${{\hat{X}}}$, and that the measurement outcome yields the value $x_l$, $l\ne k$, for which the probability would have been very small. In this case, the interpretation of the event is the obvious one: a rare event has occurred. Now suppose instead that the state of mind is very close to one of the ‘certain’ states $|x_k\rangle$, in a situation where there is a correct choice to be made (for example, in deciding what had actually happened at an event in the past—as opposed to choices for which there need not be ‘correct’ outputs). In this case, if the correct choice happens to be $|x_l\rangle$, $l\ne k$, and if the outcome $x_k$ is nonetheless chosen (even though objectively the correct choice should have been $x_l$), this evidently does not mean that an unlikely event had occurred. Rather, it means that the initial state of mind was a misguided one. Putting the matter differently, while a state of a quantum system represents the physical reality of the system, a state of mind merely represents the person’s perception of the state of the world. This difference between objective and subjective probabilities has important implications discussed below. There is also the suggestion that the state of a quantum system itself is entirely subjective^35^, but this idea will not be explored here.

The subjective nature of psychological states gives rise to the following challenge. In psychology, it is not uncommon for a group of people having varying dispositions to be given some information (e.g., an article to read or a video clip to watch) and for their responses to be examined. An example arises in the study of confirmation bias—a bias towards information that confirms their views^36,37^. The idea is to investigate how people having diverging opinions respond differently to the ‘same’ information. The issue here is that the information content of the given message such as an article or a video clip is different to people with different opinions, even though it is an identical information source.

To explain this more concretely, consider the simple example discussed above. Suppose that the preference, or the opinion, of people on a topic is represented by the choice observable ${{\hat{X}}}$. Then the information-providing observable representing an article discussing this topic is given by ${\hat{\xi }}={{\hat{X}}}+{\hat{\varepsilon }}$. However, if the first person has the state of mind $|\psi \rangle$ and the second person $|\varphi \rangle$, then the Lüders state resulting from observing ${\hat{\xi }}$ is different. Hence, just because two people are given, say, the same article to read, to assert that they are given the same information is factually false. That is, the mutual information contained in the article about the impending decision is different to different readers. Indeed, if the observed value of ${\hat{\xi }}$ is given by $\xi$, then a calculation shows that the mutual information difference is

$$\begin{aligned} \Delta J= & {} \sum _{i} \int f(\xi -x_i) \left[ q_i \ln \left( \sum _j q_j f(\xi -x_j) \right) - p_i \ln \left( \sum _j q_j f(\xi -x_j) \right) \right] \textrm{d}\xi \nonumber \\ {}{} & {} + \sum _i \left( p_i \ln p_i - q_i \ln q_i \right) , \end{aligned}$$

where $p_i=\langle x_i|\psi \rangle ^2$, $q_i=\langle x_i| \varphi \rangle ^2$ and *f*(*y*) denotes the distribution of the noise term. One important consequence in psychology is that the various conclusions drawn from such experiments on how people’s behaviour might deviate from rational Bayesian updating require fundamental reexamination because they are not given the same information.

The subjective nature of psychological states also gives rise to a mathematical challenge. In communication theory, one is typically concerned with well-established communication channels, where the signal transmitted is assumed to represent an objective reality. Therefore, there is no ambiguity in interpreting the information-carrying time series $\{{\hat{\xi }}_t\}$. However, if different receivers were to interpret the ‘same’ message differently, and if there is a need to apply statistical analysis to the behaviour of different people, then the question arises as to which information process (called the ‘filtration’ in probability theory) one should be using for statistical analysis. To my knowledge, this situation has hardly ever been examined in the vast literature of probability and stochastic analysis.

## Limitation of classical reasoning

Thus far I have assumed, for definiteness, that all decisions are compatible. What this means is that the quantum formalism advocated here, while effective, can be reduced, if necessary, to a purely classical probabilistic formulation. It seems to me that this assumption does not fully reflect the reality, and that it is plausible that not all decisions can be made simultaneously by human brains, even if there are only a small number of decisions to be made. Indeed, there are empirical examples in behavioural psychology that strongly indicate that not all decisions or opinions are compatible^10,38,39^. If so, the observables representing these choices will not commute.

The issue with the classical updating of likelihoods based on the Bayes formula is that it is not well suited to characterise changes of context, that is, changes in the sample space—represented, for example, by an arrival of information that reveals a previously unknown alternative (see^40–42^ for discussions on contextuality in human decision making). In such a scenario, the prior probability of the new alternative is zero (because it was not even known), whereas the posterior can be nonzero. Hence, in the language of probability theory, the prior and the posterior are not absolutely continuous with respect to each other, prohibiting the direct use of the Bayes formula. In contrast, such a change of context can be modelled using incompatible observables, along with the von Neumann–Lüders projection postulate.

To see this, suppose that the prior state of mind is given by $|\psi \rangle = \sum _k c_k |x_k\rangle$ when expanded in the eigenstates of ${{\hat{X}}}$, where $c_m=0$ for some *m*, and suppose that acquisition of information takes the form ${\hat{\eta }} = {{\hat{Y}}} + {\hat{\varepsilon }}$, where ${{\hat{Y}}}$ cannot be diagonalised using the basis states $\{\!|x_k\rangle \!\}$. Then it is possible that the Lüders state ${\hat{\Pi }}_\eta |\psi \rangle /\surd { \langle \psi |{\hat{\Pi }}_\eta |\psi \rangle }$ resulting from information acquisition, when expanded in $\{\!|x_k\rangle \!\}$, is such that $c_m\ne 0$, thus circumventing the constraint of the classical Bayes formula. Therefore, in a situation whereby choice observables are not compatible, the quantum-mechanical formalism proposed here and elsewhere^11^ becomes a necessity, because the modelling of the dynamical behaviour of a person cannot be achieved using the techniques of purely classical probability. As a simple example, consider two binary (yes/no) decisions that are represented by the choice observables

$$\begin{aligned} {{\hat{X}}}=\left( \begin{array}{cc} 1 &{} 0 \\ 0 &{} -1 \end{array} \right) \quad \textrm{and} \quad {{\hat{Y}}}=\left( \begin{array}{cc} \cos \phi &{} \sin \phi \\ \sin \phi &{} -\cos \phi \end{array} \right) . \end{aligned}$$

Evidently, ${{\hat{X}}}$ and ${{\hat{Y}}}$ cannot be diagonalised simultaneously, unless $\phi =0$ (mod $2\pi$). Suppose further that the initial state of mind of a person is represented by a Hilbert space vector

$$\begin{aligned} |\psi \rangle = \left( \begin{array}{c} \cos \textstyle \frac{1}{2}\theta \\ \sin \textstyle \frac{1}{2}\theta \end{array} \right) \end{aligned}$$

for some $\theta$. Then the probability that the person giving a ‘yes’ answer to question ${{\hat{X}}}$ is $\cos ^2\frac{1}{2}\theta$; whereas if question ${{\hat{Y}}}$ were asked instead, then the likelihood of giving an affirmative answer is $\cos ^2\frac{1}{2}(\theta -\phi )$. Note that strictly speaking, according to the scheme introduced here, the state space for a pair of binary decisions is four-dimensional, if the two decisions (questions) are simultaneously considered. Here, I am interested in the effect of questions being asked sequentially, and for this purpose a two-dimensional representation suffices. Thus $|\psi \rangle$ represents an abstract state of mind for which a range of binary questions may be asked.

Now suppose that question ${{\hat{Y}}}$ is asked first, and subsequently question ${{\hat{X}}}$ is asked. Then from the projection postulate, the probability of giving a ‘yes’ answer to question ${{\hat{X}}}$, irrespective of which answer was given to the first question, is $\frac{1}{4}\left( 2+\cos (\theta )+\cos (\theta -2\phi )\right)$. For $\phi \ne 0$ this is different from the *a priori* probability $\cos ^2\frac{1}{2}\theta$ of answering ‘yes’ to question ${{\hat{X}}}$. In other words, the so-called law of total probability in classical probability theory, that the unconditional expectation of a conditional expectation equals the unconditional expectation, is not applicable when one is dealing with incompatible propositions. Similarly, if question ${{\hat{X}}}$ is asked before question ${{\hat{Y}}}$, then the probability of giving a ‘yes’ answer to question ${{\hat{Y}}}$ is $\cos ^2\frac{1}{2}\theta \cos ^2\frac{1}{2}\phi + \sin ^2\frac{1}{2}\theta \sin ^2\frac{1}{2}\phi$, which is different from $\cos ^2\frac{1}{2}(\theta -\phi )$ when $\phi \ne 0$.

This example is perhaps the simplest one to demonstrate that answers to questions can be dependent on the order in which questions are asked, if the questions are not compatible (see^43^, Appendix 2, for a discussion on the order dependence). A more elaborate construction of this kind in higher dimension is found in^39^; similarly, the use of positive operator-valued measures to analyse such order dependence is explored in^44^. In any case, violation of the law of total probability shows that this empirical phenomenon of order-dependence cannot be explained using compatible observables.

For a pair of binary choices, an attempt is made in^10^ to explain the experiment discussed in^45^. The data presented in^45^ show that when people are asked if Clinton is honest, about 50% answered ‘yes’, and if they are then asked if Gore is honest, some 60% answered ‘yes’; whereas if the order of the questions is reversed, then the figures change into 68% yes for Gore followed by 60% yes for Clinton. Note however that the explanation of this effect in^10^ is incomplete because conditional probabilities are considered therein, whereas the data in^45^ concern total probabilities. The analysis of total probabilities considered here, on the other hand, shows that by setting $\theta \approx 9\pi /20$ and $\phi \approx \pi /15$, the phenomenon reported in^45^ can be explained within a $\pm 10\%$ error margin.

## Discussion

I have illustrated how the Hilbert-space formalism used in quantum theory is highly effective in modelling cognitive psychology, in particular, its dynamical aspects. I have shown how an important feature of the dynamics associated with Bayesian updating, or equivalently with the von Neumann-Lüders projection, namely, the uncertainty-reducing trend, is made transparent in this formalism. This, in turn, provides an alternative information-theoretic perspective on the free energy principle, because of the close relation between entropy and variance in communication theory. That is, the Lüders state is the one that minimises the Kullback–Leibler divergence measure^46^ from the initial state.

One important consequence of the foregoing analysis is that states of low uncertainty are always preferred ones, irrespective of whether they represent the correct choices. Therefore, if the state of mind happens to be close to one of the false choices, then with a rational updating it is difficult to escape from this neighbourhood, since to achieve this, entropy has to increase before it can be decreased again, and this is counter to biological trends^47^. In such a situation, it appears that only the accidental effect of noise, which otherwise tends to be seen as a nuisance, can rescue the person from the false choice within a reasonable timescale, at least when all choices are compatible to each other. Hence noise can assist biological systems to conduct, in effect, a kind of simulated annealing—a closely related point of view has been put forward in the context of active inference^48^.

The situation changes once we accept the thesis of^10^ that real-world decisions are never compatible, thus making it a necessity to model cognitive behaviour within the quantum formalism. To see this, consider a pair of maximally incompatible (in the sense that their eigenvectors are maximally separated) binary decisions modelled by the pair

$$\begin{aligned} {{\hat{X}}}=\left( \begin{array}{cc} 1 &{} 0 \\ 0 &{} -1 \end{array} \right) \quad \textrm{and} \quad {{\hat{Y}}}=\left( \begin{array}{cc} 0 &{} 1 \\ 1 &{} 0 \end{array} \right) , \end{aligned}$$

and suppose that the state of mind is given by

$$\begin{aligned} |\psi \rangle = \frac{1}{\sqrt{2}} \left( \begin{array}{c} 1 \\ 1 \end{array} \right) , \end{aligned}$$

or else a state very close to $|\psi \rangle$. Since ${{\hat{Y}}}|\psi \rangle = |\psi \rangle$, this means that the state of mind in relation to decision ${{\hat{Y}}}$ is already fixed to the alternative labelled by the eigenvalue $+1$, and that the likelihood of choosing the other alternative labelled by the eigenvalue $-1$ is zero, or else very close to zero anyhow. Suppose further that the ‘correct’ choice is the one labelled by the eigenvalue $-1$ (in a situation where a correct alternative exists). The tenacious classical Bayesian behaviour^19^ then implies that providing partial information ${\hat{\eta }}={{\hat{Y}}}+{\hat{\varepsilon }}$ about the truth will have little impact. Instead, if the person is given information, not about the choice ${{\hat{Y}}}$, but about ${{\hat{X}}}$ in the form ${\hat{\xi }}={{\hat{X}}}+{\hat{\varepsilon }}$, where the magnitude of noise ${\hat{\varepsilon }}$ is small, then after acquisition of this information the state will change into one of the two possible Lüders states that can result from the observations of ${\hat{\xi }}$. These two states will be close to one of the two eigenstates of ${{\hat{X}}}$. If partial information ${\hat{\eta }}={{\hat{Y}}}+{\hat{\varepsilon }}$ is subsequently provided, then irrespective of which Lüders state is chosen, the state of mind will now transform into one that is close to the truth with a high proobability. Thus the quantum formalism opens up a new possibility that was unavailable with the classical reasoning.

Let me conclude by speculating on possible implications in artificial intelligence. If one accepts the arguments presented, for example, in references^11,12^, for the premise that the probability assignment rules of quantum theory can describe human thinking more adequately than their classical counterparts, as supported by many empirical examples, then it follows that machine learning tools based on classical probability will ultimately fail to replicate human behaviour. In principle, *artificial quantum intelligence* (here I use the phrase to mean an artificial intelligence tool based on the quantum probability rules of von Neumann and Lüders, as opposed to a more commonly adopted notion of ‘quantum artificial intelligence’ using a quantum computer to enhance classical machine learning) can be implemented on classical computers (that is, without the need to build a quantum computer). However, for such an architecture to be useful, more research is needed to uncover the meaning and the implication of incompatible decisions in cognitive psychology.

### Supplementary Information

Supplementary Information.

## Data Availability

All data generated or analysed during this study are included in this published article and its supplementary information files.
